# The Treatment of Nævi

**Published:** 1916-01-15

**Authors:** W. Knowsley Sibley

**Affiliations:** Physician to St. John's Hospital for Diseases of the Skin, London.


### THE TREATMENT OF NÆVI.

By W. KNOWSLEY SIBLEY, M.A., M.D., B.C., M.R.C.P., Physician to St. John's Hospital for Diseases of the Skin, London.

Nævi, moles, or birth-marks are caused by a congenital, local overgrowth of the cutaneous vascular tissue. Small superficial quiescent nævi occasionally disappear spontaneously. If painted from time to time with contractile collodion, or liquor plumbi subacetatis, their disappearance may be hastened. As a rule, however, stronger remedies have to be resorted to.

The results of any given treatment depend on the depth of the lesion, and can be fairly accurately judged by a skilled eye, according to the changes that occur by compressing the nævus with a glass spatula. This method gives a fair picture of the result which would be obtained from treatment by radium, short of producing an erythema. In almost all methods of treatment, more or less destruction of tissue is necessary to eradicate the defect, so considerable judgment and skill are essential to ensure a neat and hardly discernible scar.

There are several destructive agents at present in use, the most popular being carbon dioxide snow. If the nævus is a superficial one with an irregular line, the snow may be dissolved in acetone or ether and applied on a camel-hair brush, repeating at intervals of about a week. This latter method is also a useful means to gradually improve the appearance, and diminish the disfiguration caused by moles in prominent positions. The applications need only be of a few seconds' duration and repeated every few days, as soon as the obvious reaction has disappeared. The freezing only being very superficial, no blebs will be produced, and if the treatment is continued sufficiently long, a considerable improvement will be appreciable. If, however, the lesion is a deeper one, more especially if the surface is raised above the level of the skin and is of a verrucoid nature, the freezing must be applied much deeper with a solid stick of the snow, moulded by pressure to the size and shape of the patch and applied with firm pressure for from thirty seconds to two or more minutes, according to the depth of tissue it is necessary to destroy.

Another useful and comparatively painless application is trichloracetic acid, just enough water being added to dissolve the crystals. This can be painted on the lesion with a camel-hair brush. In a few days a dry eschar forms, which drops off in a week or two, revealing an improvement in the lesion. This process may be repeated from time to time if necessary, and will be found satisfactory both in the case of infants and adults. If the lesion is a hairy mole, the hairs should first be removed individually by electrolysis, the result of which treament often produces a marked diminution of the birth-mark. If it does not, then one of the above methods may be resorted to.

If the lesion is quite a small one, the negative electrolysis needle may be inserted in the centre, and one to two milliampères of current passed for a few seconds to a minute or so. If a larger lesion has to be treated, both needles may be inserted, the positive in the centre, and the negative at various points in the periphery. It is well to guard the positive needle with insulating material such as shellac up to an eighth of an inch of its tip.

High-frequency currents with the point electrode may also lessen a port-wine stain after repeated treatments. Appropriate applications of radium have in many cases produced most excellent results.

